# Finite element analysis of the valgus knee joint of an obese child

**DOI:** 10.1186/s12938-016-0253-3

**Published:** 2016-12-28

**Authors:** Jun Sun, Songhua Yan, Yan Jiang, Duo Wai-chi Wong, Ming Zhang, Jizhou Zeng, Kuan Zhang

**Affiliations:** 1 School of Biomedical Engineering, Capital Medical University, Beijing, 100069 China; 2 Beijing Key Laboratory of Fundamental Research on Biomechanics in Clinical Application, Capital Medical University, Beijing, 100069 China; 3 Interdisciplinary Division of Biomedical Engineering, The Hong Kong Polytechnic University, Hong Kong, 999077 China; 4 Department of Orthopedics, Beijing Luhe Hospital, Capital Medical University, Beijing, 101149 China

**Keywords:** Knee valgus, Obese child, Finite element, Contact force, Kinematics

## Abstract

**Background:**

Knee valgus and varus morbidity is at the second top place in children lower limb deformity diseases. It may cause abnormal stress distribution. The magnitude and location of contact forces on tibia plateau during gait cycle have been indicated as markers for risk of osteoarthritis. So far, few studies reported the contact stress and force distribution on tibial plateau of valgus knee of children.

**Methods:**

To estimate the contact stresses and forces on tibial plateau of an 8-year old obese boy with valgus knee and a 7-year old healthy boy, three-dimensional (3D) finite element (FE) models of their left knee joints were developed. The valgus knee model has 36,897 nodes and 1,65,106 elements, and the normal knee model has 78,278 nodes and 1,18,756 elements. Paired t test was used for the comparison between the results from the 3D FE analysis method and the results from traditional kinematic measurement methods.

**Results:**

The p value of paired t test is 0.12. Maximum stresses shifted to lateral plateau in knee valgus children while maximum stresses were on medial plateau in normal knee child at the first peak of vertical GRF of stance phase. The locations of contact centers on medial plateau changed 3.38 mm more than that on lateral plateau, while the locations of contact centers on medial plateau changed 1.22 mm less than that on lateral plateau for healthy child from the first peak to second peak of vertical GRF of stance phase.

**Conclusions:**

The paired t test result shows that there is no significant difference between the two methods. The results of FE analysis method suggest that knee valgus malalignment could be the reason for abnormal knee load that may cause knee problems in obese children with valgus knee in the long-term. This study may help to understand biomechanical mechanism of valgus knees of obese children.

## Background

The knee joint is one of the most important and complicated joints, serving as the junction for activities of human lower extremities [1]. Knee valgus and varus morbidity is at the second top place in children lower limb deformity diseases, and can cause abnormal gait function [2]. It is known that knee valgus can cause malalignment, while malalignment raises the risk of knee osteoarthritis. Damage from mechanical stress without complete self-repair is the primary cause of osteoarthritis. The stress sources include misalignments of bones, mechanical injury and excess body weight et al. [3]. However, few studies report the contact stress and force distribution on tibial plateau of valgus knee of children.

Given the complicated anatomical structure and the variety of movement patterns of the knee joint, it is not sufficient to delineate the detailed biomechanics of the individual knee joint only using experimental methods [4]. The finite element (FE) analysis method is considered as a useful tool to predict stress and strain in complicated systems in biomechanics and bioengineering [5]. Since Brekelmans et al. [6] applied the FE analysis method to orthopedic research in 1972, it has become an important tool in the study of biomechanics [6]. Bendjaballah et al. applied computed tomography (CT) image and measurement methods to construct the knee joint FE model in 1997 [4]. Peña et al. [7] employed CT image and magnetic resonance (MR) image to create a more complete 3D model including knee ligaments in 2006, and Guo et al. [1] used FE analysis method to simulate knee joint stress in gait cycle in 2009 [1, 7]. In recent years, some researchers constructed FE models of bones and soft tissues only using MR images [8–10]. Some researchers use healthy adult lower limb model to study the knee injury mechanisms and some others focused on how the injury will affect the knee [11–13]. All these studies above focused on the adult subjects by comparison with the other researchers’ findings.

Lancianese et al. [14] employed 3D FE analysis method and kinematic measurement method to investigate the stresses in the proximal tibias of overweight children knees [14]. Their results have demonstrated that the patterns of total force distribution on proximal tibia of overweight children were different from those of normal weight children. However, only tibia and tibial cartilage was included in their concise FE model, and their results were not effectively evaluated.

Changes in contact forces (CF) and contact centers on the cartilage articulating surface have been indicated as important markers in the prevention/initiation/progression or alternatively in the evaluation of treatment stages of joint disorders [15]. The magnitude and location of CF on tibia plateau during gait cycle have been considered as markers for risk of osteoarthritis [15]. Engel et al. reported that the valgus alignment might lead to higher loading of the lateral compartment, and the unequal load distributions may result in an accelerated progression of cartilage degeneration within the knee joint [16]. There is still lack of studies to examine the CF and contact centers on tibial plateau of children, especially obese children with valgus knee. The magnitude and location of CF on tibial plateau can not be identified only by the 3D motion capture system methods, and the FE analysis methods provide an alternative way to explore the problem.

This study was designed to analyze the CF and their locations on tibial plateau of an obese child with valgus knee and a healthy child by FE analysis methods. The results from FE model were compared with results from kinematic method using 3D motion capture system and a force plate.

## Methods

An obese boy with valgus knee [age: 8 years; height: 1.41 m; weight: 47 kg; body mass index (BMI): 23.64 kg/m^2^] and a healthy boy (age: 7 years; height: 1.30 m; weight: 28.5 kg; BMI: 16.86 kg/m^2^) participated in this study. BMI > 20.4 is classified as obesity based on Group of China Obesity Task Force for children with age of 7~8 [17]. The femur-tibia angle of the left valgus knee from the obese child is 13°. A 64-layer screwing CT machine (Somatom Sensation Cardiac 64, Siemens Corporation, Germany) was used to obtain the CT scan images of the knee valgus child’s left knee. A 3.0T MR machine (United Imaging uMR770, Shanghai, China) was used to obtain the MR scanning images of the healthy child’s left knee. The kinematic data of the lower limb and the ground reaction forces (GRF) during walk were recorded simultaneously by a 3D motion capture system (Qualisys, Sweden) and a force plate (Kistler Corporation, Switzerland). Sample rates were 200 Hz for the motion capture system and 1000 Hz for the force plate. All the system was calibrated before test.

### Kinematic measurement methods

**Fig. 1:**
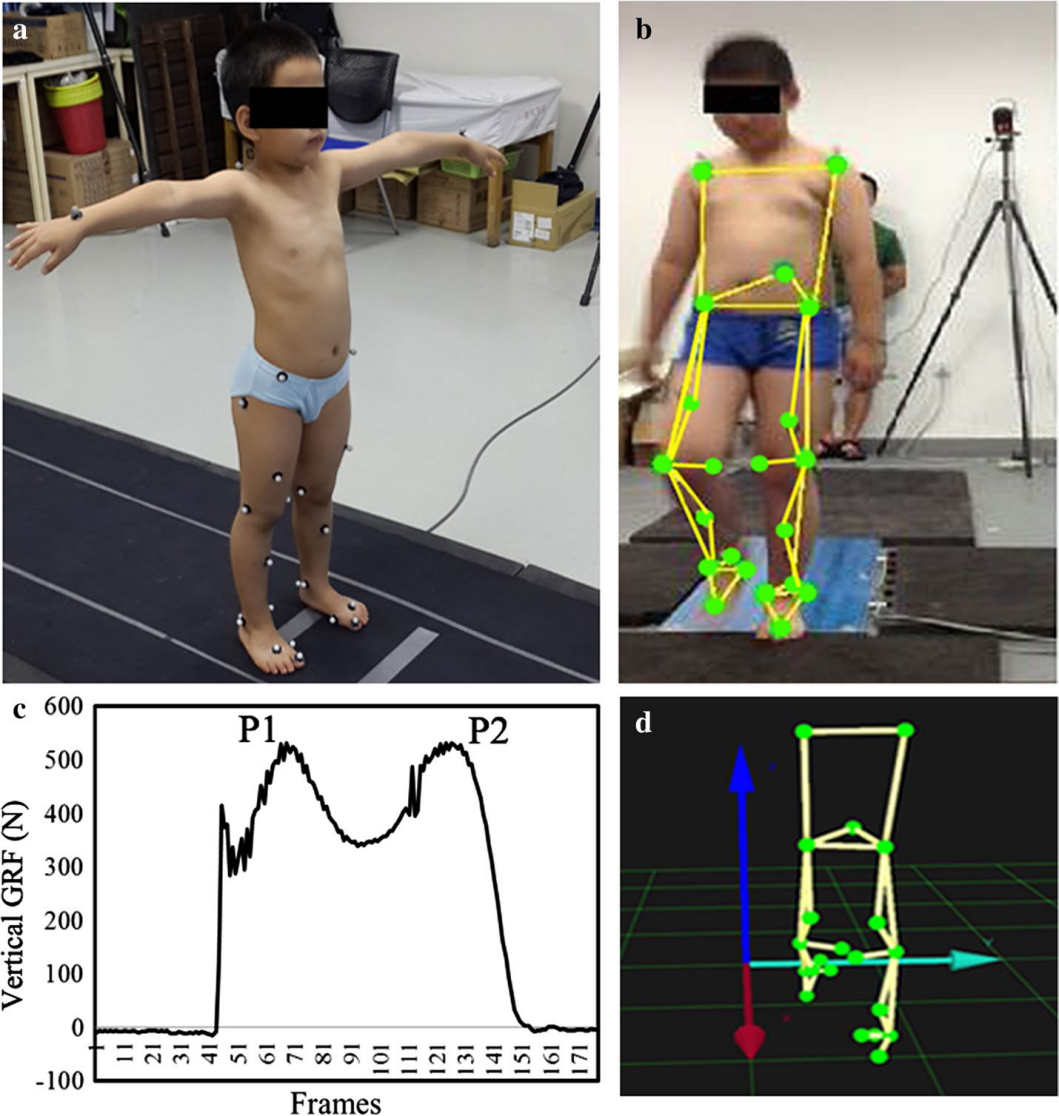
Kinematic measurement experiments using motion capture system and force plate. **a** A healthy subject under static stance situation. **b** An obese knee valgus subject under walking condition. **c** and **d** Shows the data collection progress. P1 and P2 in *figure* **c** are the first peak and second peak of vertical GRF

Twenty-one markers were attached to the skin and six motion capture cameras were set to record their motion. According to Helen-hyes model, two markers were fixed on the right and left anterior superior iliac spines. One other marker is placed on superior aspect at L5-sacral interface. Other twelve markers were placed on the following locations: the medial/lateral femoral condyle and medial/lateral ankle for both legs; the space between the second and third metatarsal heads of both feet; the right and the left heels. Four markers were placed on midthigh and midshank for both legs. These 19 markers were used for measuring the lower extremity kinematics. In addition, two other markers were fixed on shoulder joints to observe the participants’ body movement (Fig. 1).

Before the collecting data, the subjects were instructed to walk several times across the force plate to get familiar with the entire experiments. At beginning, they stepped on the force plate and kept static stance for 30 s. Then they walked on a 5-m long walkway in the gait laboratory across the force plate with natural speed. After starting recording data, we kept three trails at least when the children stepped on the force plate with their left feet. The subjects finished all the experimental procedures with bare foot. The natural speed is 1.023 ± 0.116 m/s for the knee valgus child, and 0.701 ± 0.048 m/s for the healthy child.

**Fig. 2:**
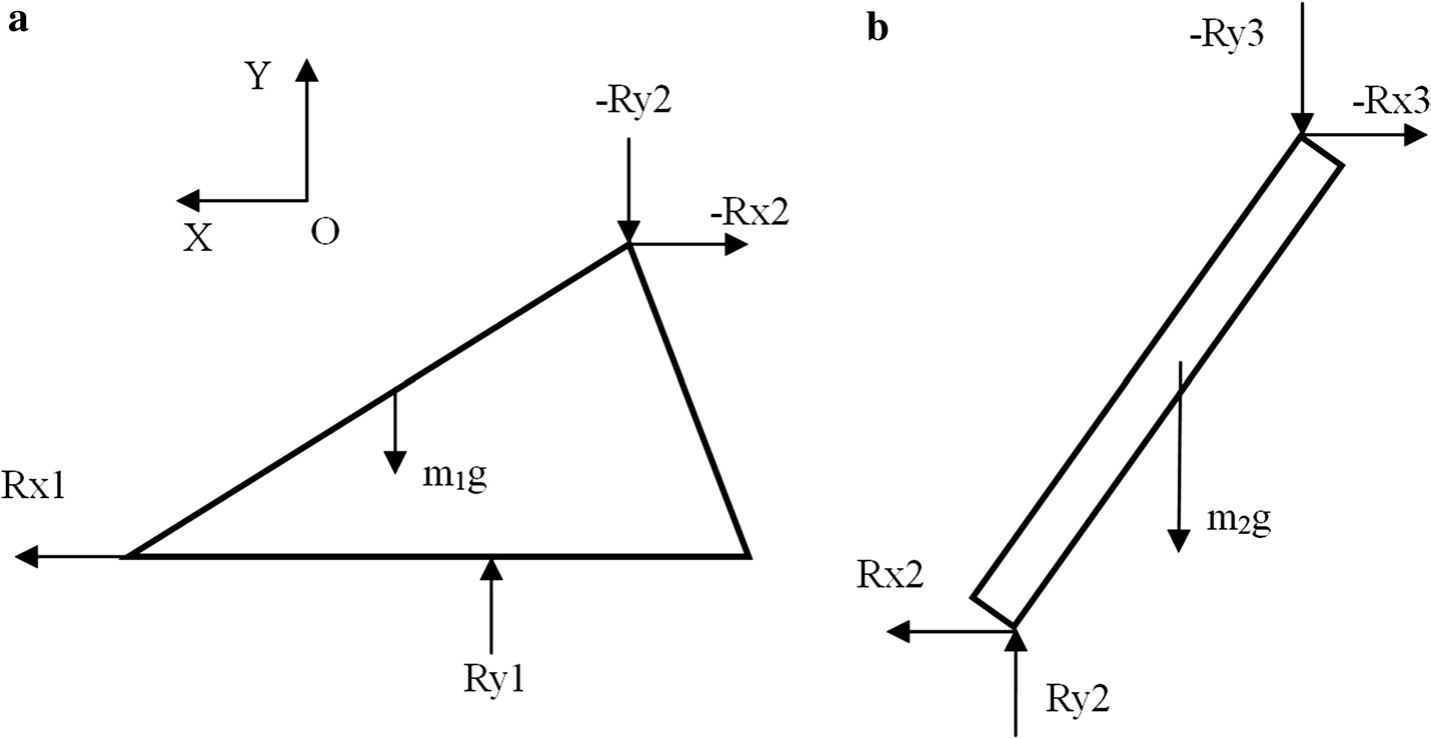
Force decomposition diagram. Force Rx_1_, Ry_1_ are the force components of ground reaction forces recorded by the force plate. **a** Shows the foot and *figure* **b** shows the calf

Inverse dynamics method was used to calculate the equivalent forces applied at the knee joint and the ankle joint during stance and walking [18, 19]. The first and second peaks of vertical GRF of stance phase were examined. Based on the force analysis diagram (Fig. 2), the translational kinetic equations were derived as followed:

1 $${\text{Rx}}_{ 1} = {\text{Rx}}_{ 2} + {\text{m}}_{ 1} {\text{a}}_{{ 1 {\text{c}}}}{\text{x}} ,$$

2 $${\text{Ry}}_{ 1} = {\text{ Ry}}_{ 2} + {\text{ m}}_{ 1} {\text{g }} + {\text{ m}}_{ 1} {\text{a}}_{{ 1 {\text{c}}}}{\text{y}} ,$$

3 $${\text{Rx}}_{ 2} = {\text{ Rx}}_{ 3} + {\text{ m}}_{ 2} {\text{a}}_{{ 2 {\text{c}}}} {\text{x}},$$

4 $${\text{Ry}}_{ 2} = {\text{ Ry}}_{ 3} + {\text{ m}}_{ 2} {\text{g }} + {\text{ m}}_{ 2} {\text{a}}_{{ 2 {\text{c}}}}{\text{y}} .$$

5 $${\text{a}}_{{ 1 {\text{c}}}} {\text{x}} = \, \left( {{\text{a}}_{\text{toe}} {\text{x}} + {\text{a}}_{\text{Heel}} {\text{x}}} \right)/ 2,$$

6 $${\text{a}}_{{ 1 {\text{c}}}} {\text{y}} = \, \left( {{\text{a}}_{\text{Toe}} {\text{y}} + {\text{a}}_{\text{Heel}} {\text{y}}} \right)/ 2,$$

7 $${\text{a}}_{{ 2 {\text{c}}}} {\text{x}} = \, \left( {{\text{a}}_{\text{Knee}} {\text{x}} + {\text{a}}_{\text{Heel}} {\text{x}}} \right)/ 2,$$

8 $${\text{a}}_{{ 2 {\text{c}}}} {\text{y}} = \, \left( {{\text{a}}_{\text{Knee}} {\text{y}} + {\text{a}}_{\text{Heel}} {\text{y}}} \right)/ 2,$$

It is assumed that the x and y components of foot and calf’s accelerations are towards OX and OY directions. According to Leva et al. the foot and calf’s weight account for 1.37 and 4.33% of body weight, respectively [20]. At the first/second peak of vertical GRF of stance phase, the acceleration a_toe_x, a_toe_y, a_heel_x, a_heel_y and a_knee_x, a_knee_y are the acceleration components of the toe, heel and knee, which are recorded by Qualysis system. The a_1c_x and a_1c_y are the acceleration components of foot, while the a_2c_x and a_2c_y are the acceleration components of shank. Rx_1_, Ry_1_ are force Fx, Fz of GRF recorded by force plate (Figs. 1, 2). Force Rx_2_ and Ry_2_ are the force components burdened by ankle. Force Rx_3_ and Ry_3_ are the force components of knee calculated by Qualysis.

### Finite element model

The 3D FE model of valgus knee was constructed using CT image, while the normal knee model was constructed using MR images. The scanning layer thickness was 1.0 mm, and the distance between layers was 1.0 mm. The scope of the scan was from the distal femur to proximal tibia.

**Fig. 3:**
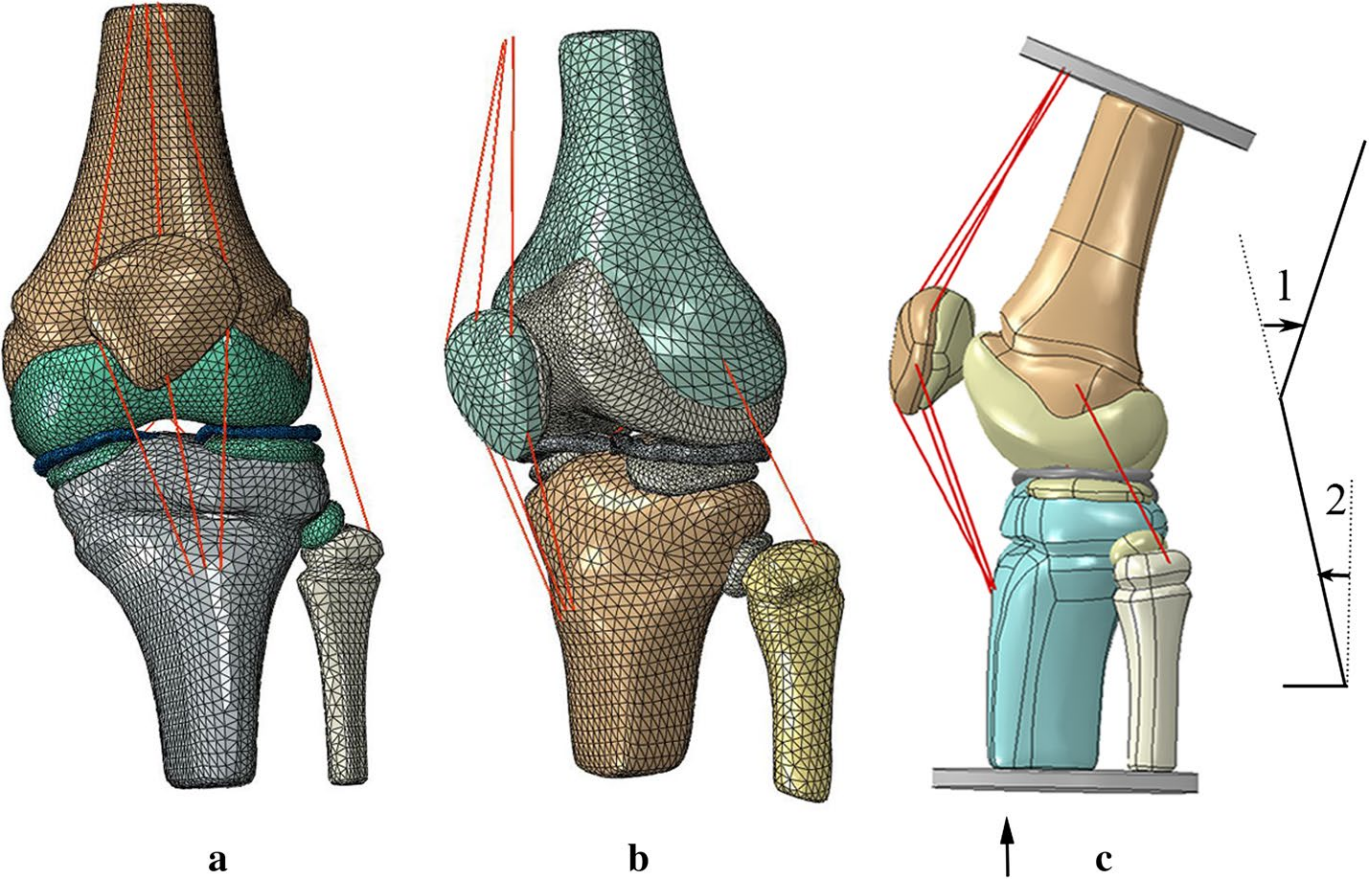
Finite element model and model geometry of the knee joints. **a** Finite element model of the knee valgus child’s left knee. **b** Finite element model of the healthy child’s left knee. **c** The model geometry of natural walk with knee flexion angle *1* and ankle flexion angle *2*. Loads were applied on the distal end of the tibia and fibula

The bony structure and soft tissue boundaries in CT and MR images of the knee joint were identified and segmented using MIMICS v16.0 (Materialise, Leuven, Belgium). The meniscus, tibial cartilage, femoral cartilage, collateral ligaments and anterior/posterior cruciate ligaments were reconstruction based on the images and other studies [1, 4, 11, 21]. The 3D model was then imported into Rapidform XO3 (Rapidform corporation, USA) to reduce noise. The solid model was assembled and meshed into 3D 4-node tetrahedral elements using ABAQUS v6.13-4 FE package (Hibbitt, Karlsson and Sorensen, Inc., Pawtucket, RI) to create the final FE model and conduct the engineering analysis (Fig. 3). The model of the valgus knee joint contains 36,897 nodes and 1,65,106 elements, and the model normal knee joint has 78,278 nodes and 1,18,756 elements. The results of convergence test for the element size showed that the errors were below 10%. As the inertia affects not significant at the stance phase of gait [22], it was neglected for the simplification of problems while quasi-static analysis was applied in the FE model.

All materials were assumed isotropic, homogeneous and linearly elastic for the purpose of analyzing contact stresses [21]. The elastic modulus and Poisson ratio are shown in Table 1 [11, 13, 14, 23–28]. Femoral cartilage with tibia cartilage and femoral cartilage with meniscus were considered as frictionless surface to surface contacts including finite sliding. The other contacts were applied as tied contacts to simulate the junction of the knee joint [1, 13]. The proximal end of the femur was fixed and the tibia and fibula bear the load of Ry_2_. A plate was added on the distal end of the tibia and fibula. Knee joint angle and ankle joint angle were set according to kinematic results for natural walk. Contact center locations were measured using maximum strain method [29]. The distribution and nephogram of von-Mises stresses and contact stresses on tibia plateau were calculated.

**Table 1:** Material parameters of the finite element model

Structure	Elastic modulus (MPa)	Poisson’s ratio	References
Femur	17,000	0.3	Kim et al. [23]
Tibia	12,200	0.3	Limbert et al. [24], Lancianese et al. [14]
Fibula	15,500	0.24	Berteau et al. [25]
Patella	15,000	0.3	Kiapour et al. [11]
Cartilage	5	0.46	Li et al. [26]
Meniscus	59	0.49	LeRoux et al. [27]
Ligament	6	0.4	Siegler et al. [28], Peña et al. [13]

The paired t test was used to compare the results between the traditional kinematic measurement method and the 3D FE analysis method. The statistical analysis was analyzed by software SPSS 21.0.

## Results

For the knee valgus child, the knee joint CF (Ry_3_) calculated from the traditional kinematic measurement method during static stance was 204.04 N, and it was 221.08 N (187.16 N on medial plateau cartilage and 33.92 N on lateral plateau cartilage) from the FE analysis method (Table 2). The maximum von-Mises stresses were 0.95 MPa on medial plateau, and 0.35 MPa on lateral plateau. The maximum contact stresses were 2.19 MPa on medial plateau, and 0.74 MPa on lateral plateau (Table 3). The Rx_1_ and Ry_1_ of GRF were −4.04 ± 36.72 N and 496.89 ± 35.16 N at the first peak of vertical GRF of stance phase; and were −6.40 ± 4.98 N and 533.63 ± 4.91 N at the second peak. The total knee CF (Ry_3_) were 470.60 ± 34.98 N at the first peak and 505.66 ± 2.90 N at the second peak by the kinematic measurement method while Ry_3_ were 484.06 ± 35.20 N (78.73 ± 10.00 N on medial cartilage; 405.33 ± 25.87 N on lateral cartilage) and 521.20 ± 7.34 N (500.60 ± 4.34 N on medial cartilage; 20.60 ± 3.02 N on lateral cartilage) respectively by the FE model (Table 2).

**Table 2:** Total contact forces (CF) on tibial plateau calculated from FE analysis method and kinematic method

	Ry_1_ (N)	Ry_3_ (N)	Total CF (N)	Ratio	Medial CF (N)	Lateral CF (N)
Valgus stance	230.3	204.04	221.08	0.92	187.16	33.92
Valgus peak 1	496.89 ± 35.16	470.60 ± 34.98	484.06 ± 35.20	0.97 ± 0.01	78.73 ± 10.00	405.33 ± 25.87
Valgus peak 2	533.63 ± 4.91	505.66 ± 2.90	521.20 ± 7.34	0.97 ± 0.02	500.60 ± 4.34	20.60 ± 3.02
Control stance	135.82	123.73	121.81	0.98	26.68	95.13
Control peak 1	264.50 ± 5.85	241.22 ± 4.28	243.54 ± 10.13	0.99 ± 0.02	68.83 ± 2.69	174.71 ± 7.44
Control peak 2	282.47 ± 11.69	254.63 ± 9.25	263.91 ± 12.12	0.97 ± 0.01	140.87 ± 6.40	123.04 ± 5.76

Ry_1_ is the vertical ground reaction force (GRF) and Ry_3_ is the knee joint force calculated by the traditional kinematic measurement methods. The total CF represents the contact forces calculated by the finite element analysis method. The ratios were obtained from ‘Ry_3_/Total CF’. The total CF is consisted of the CF on medial tibial plateau and the CF on lateral tibial plateau

**Table 3:** Maximum von-Mises stresses and maximum contact stresses of the tibial plateau of different conditions

	von-Mises stresses/medial	von-Mises stresses/lateral	Contact stresses/medial	Contact stresses/lateral
Valgus stance	0.95	0.35	2.19	0.74
Valgus peak 1	0.75 ± 0.06	2.22 ± 0.14	1.24 ± 0.10	4.77 ± 0.28
Valgus peak 2	1.64 ± 0.06	0.46 ± 0.04	3.07 ± 0.02	0.80 ± 0.09
Control stance	1.04	1.82	1.68	3.77
Control peak 1	3.57 ± 0.02	2.52 ± 0.06	4.85 ± 0.06	4.11 ± 0.04
Control peak 2	3.44 ± 0.37	3.16 ± 0.51	6.65 ± 0.17	5.56 ± 0.11

All units of data in the form are MPa

The knee angle and ankle angle were 22.29° ± 1.15° and 1.66° ± 8.00° at the first peak of vertical GRF during stance phase, and were 9.41° ± 6.25° and 18.14° ± 6.51° at the second peak. At the first peak, the maximum von-Mises stresses were 0.75 ± 0.06 MPa on medial plateau and 2.22 ± 0.14 MPa on lateral plateau, while the maximum contact stresses of tibial plateau were 1.24 ± 0.10 MPa on medial plateau and 4.77 ± 0.28 MPa on lateral plateau. At the second peak, the maximum von-Mises stresses were 1.64 ± 0.06 MPa on medial plateau, 0.46 ± 0.04 MPa on lateral plateau, while maximum contact stresses of tibial plateau were 3.07 ± 0.02 MPa on medial plateau, 0.80 ± 0.09 MPa on lateral plateau (Table 3).

For the healthy child, the knee joint force (Ry_3_) was 123.73 N by traditional kinematic measurement method, and was 121.81 N (26.68 N on medial cartilage and 95.13 N on lateral cartilage) by FE analysis method (Table 2). The maximum von-Mises stresses were 1.04 MPa on medial plateau, and 1.82 MPa on lateral plateau. The maximum contact stresses were 1.68 MPa on medial plateau, and 3.77 MPa on lateral plateau (Table 3). The Rx_1_ and Ry_1_ were −3.17 ± 21.55 N and 264.50 ± 5.86 N at the first peak of vertical GRF of stance phase and were 5.68 ± 20.18 N and 282.47 ± 11.70 N at the second peak. Ry_3_ were 241.22 ± 4.28 N at the first peak and 254.63 ± 9.25 N at the second peak by the traditional kinematic measurement method, while Ry_3_ were 243.54 ± 10.13 N (68.83 ± 2.69 N on medial cartilage; 174.71 ± 7.44 N on lateral cartilage) and 263.91 ± 12.12 N (140.87 ± 6.40 N on medial cartilage; 123.04 ± 5.76 N on lateral cartilage) respectively by the FE model (Table 2).

The knee angle and ankle angle were 16.60° ± 4.28° and 6.80° ± 1.81° at the first peak of vertical GRF, and were 19.27° ± 1.82° and 18.87° ± 1.91° at the second peak. At the first peak, the maximum von-Mises stresses were 3.57 ± 0.02 MPa on medial plateau, and 2.52 ± 0.06 MPa on lateral plateau, while the maximum contact stresses of tibial plateau were 4.85 ± 0.06 MPa on medial plateau, and 4.11 ± 0.04 MPa on lateral plateau. At the second peak, the maximum von-Mises stresses were 3.44 ± 0.37 MPa on medial plateau, and 3.16 ± 0.51 MPa on lateral plateau, while maximum contact stresses of tibial plateau were 6.65 ± 0.17 MPa on medial plateau, and 5.56 ± 0.11 MPa on lateral plateau (Table 3).

The p-value of paired t test is 0.12 (>0.05) after adjusted the weight of calf. All the consistencies of results from two methods are greater than 0.92.

**Fig. 4:**
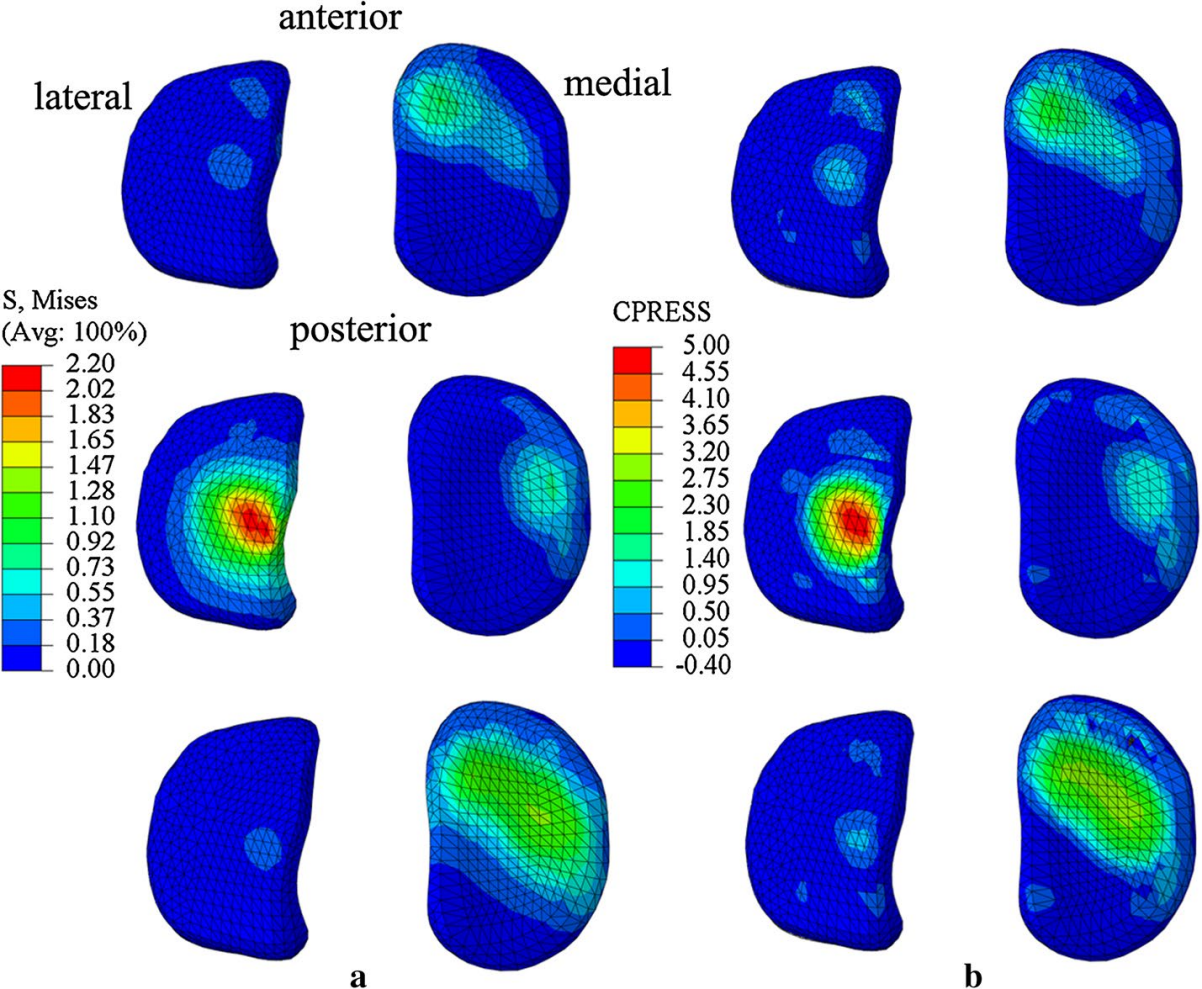
Nephograms of von-Mises stresses and contact stresses of valgus knee model. From *top* to *bottom* are results of static stance, first peak and second peak of vertical GRF of stance phase during natural walk for valgus knee model. **a** Shows nephograms of von-Mises stresses. **b** Shows nephograms of contact stresses

**Fig. 5:**
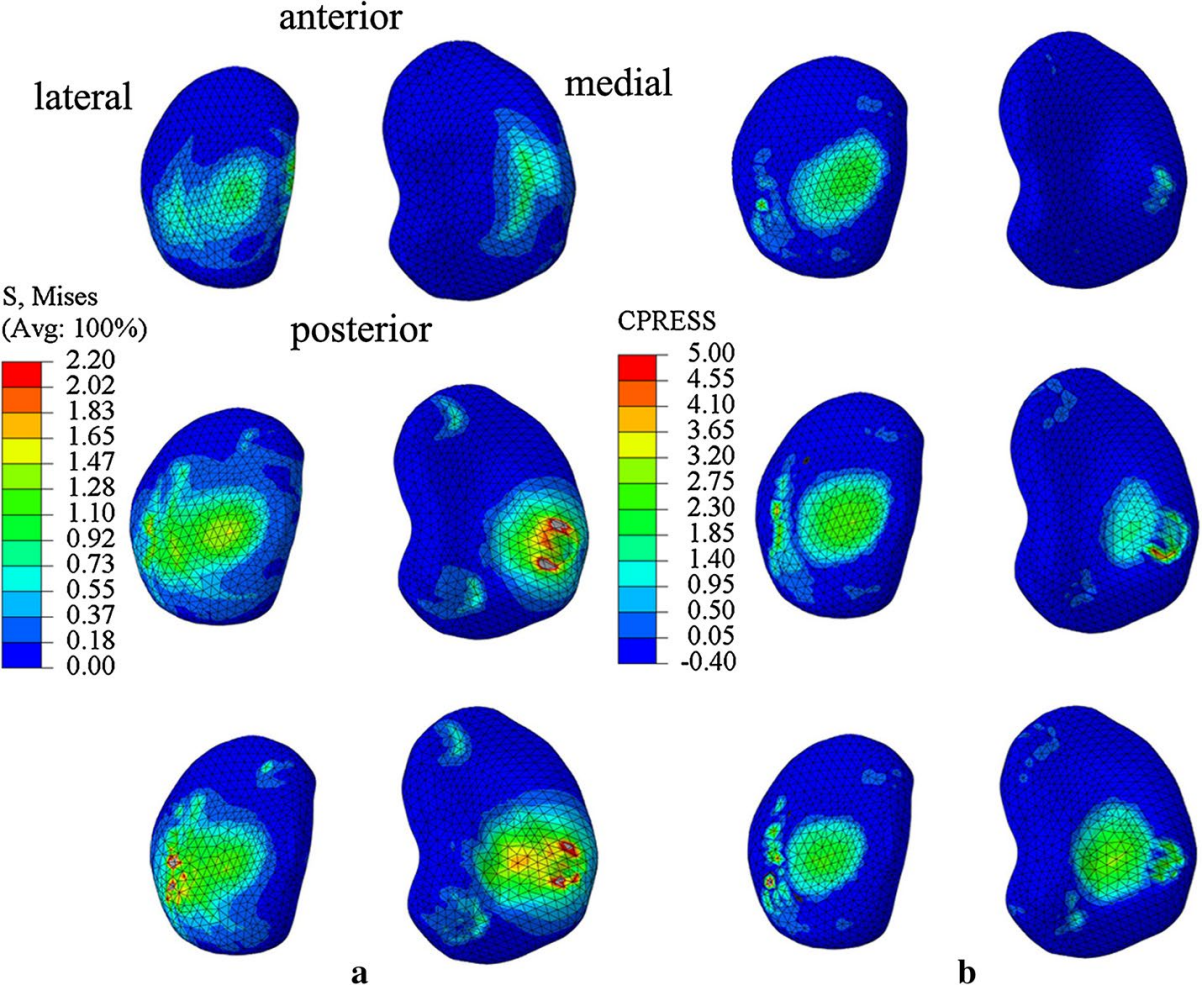
Nephograms of von-Mises stresses contact stresses and of normal knee model. From *top* to *bottom* are results of static stance, first peak and second peak of vertical GRF of stance phase during natural walk for normal knee model. **a** Shows nephograms of von-Mises stresses. **b** Shows nephograms of contact stresses

For the valgus knee, the average locations of contact centers were 27.70 mm for medial cartilage and 12.95 mm for lateral cartilage from the tibia plateau center at the first peak of vertical GRF of stance phase in medial–lateral direction, and were 24.32 and 12.95 mm at the second peak. For the normal knee, the average contact centers were 19.26 mm for medial cartilage and 12.50 mm for the lateral cartilage from the tibial plateau center at the first peak, and were 18.51 and 14.47 mm at the second peak (Table 4). The tibia plateau center was defined as the center of medial–lateral direction and anterior-posterior direction. The nephograms of von-Mises stresses and contact stresses were showed in Figs. 4 and 5.

**Table 4:** Locations of contact centers in medial–lateral direction at the first/second peak of vertical GRF

	Medial contact center (mm)	Lateral contact center (mm)	Medial contact forces (%)	Lateral contact forces (%)
A1/A2	27.70/24.32	12.95/12.95	16.22/96.05	83.78/3.95
B1/B2	19.26/18.51	12.50/14.47	28.26/53.38	71.74/46.62
C1/C2	24.50/23.50	11.50/16.00	74.54/78.93	25.46/21.07
D1/D2	21.50/19.50	12.0/12.50	76.97/81.00	23.03/19.00

A1/A2, B1/B2 represent the locations of contact centers at the first/second peak of vertical GRF for valgus knee model and normal knee model in this study. C1/C2, D1/D2 represent the adult normal knee and osteoarthritis knee model results at 25 and 75% of stance phase in Marouane’s study [29]

## Discussion

The CF calculated from FE analysis method were consistent with the forces calculated from the 3D kinematic measurement method in both valgus knee and normal knee. The maximum contact stresses and von-Mises stress shifted to lateral plateau at the first peak of vertical GRF of stance phase in valgus knee model. However, the maximum contact stresses were on medial plateau at the first peak in the normal knee model in this study. Previous studies for normal adult subjects have shown that maximum contact pressures were on medial plateau, which means that the abnormal distribution of stresses on tibial plateau was possibly caused by knee valgus [1, 29].

In our study, the forces on the medial side and the forces on the lateral side at the first peak were 16.22 ± 0.01 and 83.78 ± 0.01% of total CF in valgus knee model, and were 96.05 ± 0.01 and 3.95 ± 0.01% for second peak (Table 4). The pattern of force distribution was the similar with the results from Lancianese et al. [14] that the CF on proximal tibia were less on medial side (47.39%) than on lateral side (52.61%) for overweight children with valgus knee at the first peak of vertical GRF of stance phase. The differences of percentages of the two studies might be because of different knee valgus angles and different BMI condition. The average BMI of Lancianese’s subjects were 17.9 ± 2.2 kg/m^2^, while the BMI of the knee valgus subject in this study was 23.64 kg/m^2^. However, the knee valgus angle and nephograms about distribution of contact stresses were not provided in Lancianese’s study. The results from a FE knee model for a healthy adult have shown that 74.54 and 25.46% of total CF are on medial plateau and on lateral plateau at 25% period of stance phase, and 78.93 and 21.07 at 75% period of stance phase [29]. It indicates that the CF of proximal tibia is more on medial side than on lateral side for healthy subjects. In contrast, the pattern of forces distribution of normal knee in this study was that the forces on medial plateau and on lateral plateau were 28.26 ± 0.01 and 71.74 ± 0.01% at the first peak of stance phase, and were 53.38 ± 0.01 and 46.62 ± 0.01% at the second peak of stance phase. The possible reason for the differences between our study and Lancianese’s study might be the different ages and different velocities. The ages of subjects were 12.2 ± 1.6 years, and average velocity is 1.20 m/s in Lanecianese’s study [14]. The age of the healthy subject in our study is 7 years, and the average velocity is 0.701 m/s. Results from our study has shown that the CF shifted laterally much more in valgus knee model than those in normal knee model at the first peak of vertical GRF of stance phase. The possible reason is that in order to keep body stable, the center of gravity has to be shifted laterally when the body is decelerating at the first peak of vertical GRF of stance phase. Considering the structure of valgus knee and the excessive body weight, the lateral tibial plateau bears more forces at the first peak than at the second peak, because the center of gravity started to shift to the other limb at the second peak.

The locations of contact centers shifted laterally for 3.38 mm on medial plateau and 0 mm on lateral plateau from first peak to second peak of vertical GRF of stance phase for the valgus knee model, while it shifted laterally 0.75 and 1.97 mm for normal knee model in this study. Marouane et al. [29] reported that the locations of contact centers moved laterally about 2.00 mm on medial plateau and 0.05 mm on lateral plateau from 25% period to 75% period of stance phase from osteoarthritis knee model, but 1.00 and 4.50 mm from normal adult knee model (Table 4). The pattern of changes of locations in the valgus knee model is similar to that in the osteoarthritis knee model by Marouane et al. [29]. The locations of contact centers changes more on medial plateau than on lateral plateau in the valgus knee model while the locations of contact centers changes more on lateral plateau than on medial plateau in normal knee model.

The subject in this study bears excessive body weight with lower limb malalignment. The results about the abnormal force distribution of the valgus knee of child could cause potential medical problems in the long term. More studies should be carried out to focus on the development of valgus knee of children especially for obese children in the future.

There are some limitations to present study. First, we chose the first and second peak of vertical GRF during stance phase, which could be generalized to the other period of stance phase. Second, westerners’ or adults’ material mechanical parameters were applied in this study, which might cause influence on results because of race differences and age differences. Besides, quasi-static analysis was applied in the FE model with neglecting the inertia, which may not be ideal. Lastly, only two subjects (one with valgus knee and one with normal knee) were included in this study, and more subjects should be recruited for related studies in the future if possible.

## Conclusions

The results from this study have shown the obvious differences of mechanical properties between the valgus knee and the normal knee. Maximum stresses shifted lateral plateau in knee valgus children while maximum stresses were on medial plateau in normal knee children at the first peak of vertical GRF of stance phase. The locations of contact centers on medial plateau change more than that on lateral plateau, while the locations of contact centers on medial plateau changed less than that on lateral plateau for healthy child from the first peak to second peak of vertical GRF of stance phase. It suggests that valgus knee could be the reason for abnormal knee load that may cause knee problems in obese children with valgus knee in the long term. This study may help to understand biomechanical mechanism of valgus knees of obese children.

## Abbreviations

3D: three dimensional
FE: finite element
CT: computed tomography
MR: magnetic resonance
CF: contact force
BMI: body mass index
GRF: ground reaction force
